# Sex-Specific Sociodemographic Correlates of Dietary Patterns in a Large Sample of French Elderly Individuals

**DOI:** 10.3390/nu8080484

**Published:** 2016-08-08

**Authors:** Valentina A. Andreeva, Benjamin Allès, Gilles Feron, Rebeca Gonzalez, Claire Sulmont-Rossé, Pilar Galan, Serge Hercberg, Caroline Méjean

**Affiliations:** 1 Equipe de Recherche en Epidémiologie Nutritionnelle (EREN), Centre de Recherche en Epidémiologie et Statistiques COMUE Sorbonne-Paris-Cité, UMR Université Paris 13/Inserm U1153/Inra U1125/Cnam, Bobigny 93017, France; b.alles@eren.smbh.univ-paris13.fr (B.A.); r.gonzalez@eren.smbh.univ-paris13.fr (R.G.); p.galan@eren.smbh.univ-paris13.fr (P.G.); s.hercberg@eren.smbh.univ-paris13.fr (S.H.); c.mejean@eren.smbh.univ-paris13.fr (C.M.); 2 Centre des Sciences du Goût et de l’Alimentation, CNRS, Inra U1324, Université de Bourgogne, Dijon 21000, France; gilles.feron@dijon.inra.fr (G.F.); Claire.Sulmont@dijon.inra.fr (C.S.-R.); 3 Département de Santé Publique, Hôpital Avicenne, Bobigny 93017, France

**Keywords:** dietary patterns, elderly, sociodemographic factors, general population

## Abstract

This cross-sectional analysis provides up-to-date information about dietary patterns (DP) and their sociodemographic correlates in European elderly individuals. We studied 6686 enrollees aged 65+ (55% women) in the ongoing French population-based NutriNet-Santé e-cohort. Diet was assessed via three 24 h records. The sex-specific correlates of factor analysis derived DP were identified with multivariable linear regression. Using 22 pre-defined food groups, three DP were extracted. The “healthy” DP (fruit, vegetables, grains, nuts, fish) was positively associated with education, living alone, and being a former smoker (women), and negatively associated with being overweight, current smoker (men), age 75+ years, having hypertension, and obesity (women). The “western” DP (meat, appetizers, cheese, alcohol) was positively associated with BMI (men) and being a former/current smoker; it was negatively associated with age 75+ years (women) and living alone. The “traditional” DP (bread, potatoes, milk, vegetables, butter, stock) was positively associated with age and negatively associated with being a former/current smoker, education (men), and residing in an urban/semi-urban area. The findings support the diversity of DP among the elderly, highlighting sex-specific differences. The “healthy” DP explained the largest amount of variance in intake. Future studies could replicate the models in longitudinal and international contexts.

## 1. Introduction

Globalization, dietary transition (reflecting increased consumption of processed foods and convergence in dietary habits), and rapid population aging are salient features of the current worldwide context [1]. These factors are posited to increase the morbidity and mortality burden in terms of chronic diseases, including obesity [2]. Aging in particular has been associated with dental enamel erosion, decreased physical activity, and increased risk of nutritional deficiencies [3,4]. Apart from lifetime preferences and physiological changes, key determinants of diet among the elderly include living arrangements, finances, transportation, and disability [5]. Evidence suggests that older individuals adapt their diets as a consequence of comorbidity, dental problems, and olfactory deficiencies [6,7,8]. Nonetheless, marked heterogeneity in dental state, nutritional status, and dietary patterns (DP) among the elderly has been reported [9,10]. In turn, DP have been identified as important predictors of morbidity and mortality risk. It has been suggested that a DP consistent with dietary guidelines regarding consumption of fruit, vegetables, whole grains, fish, and low-fat dairy might be associated with superior nutritional status, cognitive function, quality of life, and survival among the elderly [11,12]. 

In general, evidence about the beneficial effects of nutrition on health is stronger with DP than with individual foods and/or nutrients because DP reflect the interrelations among the different food constituents [13]. Favoring dietary variety and selecting nutrient-rich foods is likewise underscored by the notion of food synergy [13,14]. In the European context, important sources of DP data in individuals aged 65+ were the EPIC-Elderly study [15] and the SENECA study [9]. Both of these cohorts, however, contain data that are over a decade old and to the best of our knowledge, no current information derived from epidemiological research exists. In France—where dietary habits are regarded as being in the middle of the north-south gradient of European diets [15] and where rates of obesity among the elderly exhibit a marked increasing trend [16]—prior evidence has suggested that even among sub-populations of elderly, DP can vary substantially [17]. Several reports—based on the Three-City study and the E3N study (the French component of the EPIC cohort) and featuring dietary data collected from the 1990s through the early 2000s—provided useful information about different DP and their correlates among French elderly [10,18,19,20,21]. However, apart from one preliminary report from a single French region [22], current evidence about dietary habits in French elderly individuals nationwide is lacking. 

Hence, the objectives of the present cross-sectional study were to provide up-to-date information about DP and their sociodemographic correlates in French elderly, using data from a large nationwide sample. In line with existing knowledge, the principal hypothesis regarding such correlates pertained to a positive association between dietary quality (i.e., high intake of fruit, vegetables, whole grains, fish) and socioeconomic status. 

## 2. Methods

### 2.1. Context and Sample

The present study is part of the ALIMASSENS Collaborative Project which was launched in France in 2014. It is a five-year multidisciplinary, multicomponent project with the ultimate objective of developing food products of high nutritional quality adapted to the masticatory capacities of non-institutionalized elderly (65+ years) [23]. The epidemiological component of the ALIMASSENS project is based on data from the ongoing NutriNet-Santé e-cohort. It was launched in France in May 2009, with enrollment and participation taking place exclusively online via a dedicated and secure web site [24]. Adults with Internet access are recruited via a combination of traditional (e.g., flyers available in doctors’ offices) and online (e.g., website advertising) strategies including vast, recurrent multimedia campaigns (television, radio, national/regional newspapers, and billboards). The provision of informed consent and an electronic signature is mandatory for enrollment. The NutriNet-Santé study was approved by the ethics committee of the French Institute for Health and Medical Research (IRB INSERM n° 0000388FWA00005831) and by the National Commission on Informatics and Liberty (CNIL n° 908450 and n° 909216). 

Upon enrollment, participants are asked to complete a set of five questionnaires: sociodemographics and lifestyle, health status, physical activity, anthropometrics, and diet [24]. For the present study, we used data from enrolled volunteers aged 65+ years, residing in metropolitan France, with at least three available 24 h dietary records (described below) provided during the first two years following enrollment.

### 2.2. Dietary Assessment

Dietary assessment in NutriNet-Santé is carried out via a user-friendly web-based 24 h dietary record tool designed for self-administration. The study features annual dietary assessment over three non-consecutive days (spread over a 2-week period), including two weekdays and one weekend. Participants report intake with the help of a food/beverage browser or a search engine. For each food and beverage item consumed over a period of 24 h (midnight to midnight), participants are asked to provide detailed information about the quantity, preparation/recipes/seasoning, and the corresponding settings (time and place). The tool features a comprehensive user’s guide and a built-in control system (with visual cues and prompts) both of which help minimize the chance of forgetting consumed items. Portion sizes can be estimated using validated photographs [25]; a published food composition table with >2000 different food items is used to estimate macro- and micronutrient intake [26]. For the present analysis, 24 h dietary records were obtained between May 2009 and December 2014. 

### 2.3. Dietary Patterns

All reported food and beverage items were initially grouped into 35 food/beverage groups (Supplementary Table S1), which were then reduced to 22 food/beverage groups, based on the absolute quantities of daily intake and the meaningful association among the different groups. Next, using factor analysis with orthogonal transformation techniques (varimax rotation), we extracted latent factors (i.e., DP) that are independent linear combinations of these food groups, thereby maximizing the explained variance in dietary intake. We determined the number of DP to be retained according to the following criteria: eigenvalues > 1.30, Cattel’s Scree test (i.e., plot of the proportion of the variance accounted for by each DP), and the logical interpretability of the factors given the food/beverage groups with the highest factor loadings [27]. Food groups with factor loadings > 0.25 were regarded as being meaningfully associated within a given DP. Since one of the food groups—“legumes/pulses”—did not show any meaningful associations with any of the DPs (all factor loadings < 0.25), it was omitted from the analysis. The food/beverage groups and corresponding DP are presented in Table 1.

### 2.4. Covariates

We used the following sociodemographic and health status data provided via self-administered questionnaires: sex, age, body weight and height, educational level, retirement status, most recent occupational category, smoking, living arrangement, residential area density, prevalent hypertension (self-report and/or antihypertensive treatment), diabetes (self-report and/or anti-diabetic treatment), and history of major cardiovascular disease (myocardial infarction, stroke, acute coronary syndrome).

### 2.5. Statistical Analysis

We used Black’s [28] method to identify energy under-reporting and such records were removed from the analysis. The sample’s sociodemographic and health status characteristics are reported as percentages from chi-squared tests or mean (SD) from Student *t* tests, as appropriate. We fit multivariable linear regression models in order to estimate the association of each DP with the following sociodemographic and health status correlates: age (divided into three categories: 65–69 years (reference), 70–74 years, 75 + years), body mass index (BMI, calculated as the weight in kg divided by the squared height, and then split into three categories: normal weight <25.0 kg/m^2^ (reference), overweight between 25.0 and 29.9 kg/m^2^, and obese ≥30.0 kg/m^2^), living arrangement (married/cohabiting = reference), educational level (up to high school = reference), smoking status (never smoker = reference), residential area density (<20,000 inhabitants = reference), and prevalent hypertension (no = reference). Specifically, the following linear model was estimated (where *y_i_* {1, 2, 3} represents each DP, β is a regression coefficient, and ε*_i_* is the error term):

*y_i_* = β_1i_*Age + β_2i_*BMI + β_3i_*Living arrangement + β_4i_*Education + β_5i_*Smoking + β_6i_*Area + β_7i_*Hypertension + ε*_i_* (1)

For these models, the DP scores were energy-adjusted using the residual method defined by Willett and Stampfer [29]. In line with evidence of sex-specific differences in dental status, total energy intake, and DP [30,31,32], we performed tests for interaction by sex and then fit sex-specific models. 

### 2.6. Supplementary Analysis

In a supplementary analysis, we also calculated an a priori score (modified Programme National Nutrition Santé-Guideline Score, mPNNS-GS) in order to augment understanding of the DP in the sample with respect to adherence to dietary guidelines, and to facilitate future cross-study comparisons. The mPNNS-GS is a diet quality score based on the French dietary guidelines (Programme National Nutrition Santé, PNNS) [33]. The non-modified version of the score additionally reflects adherence to physical activity recommendations. The maximum score of the mPNNS–GS is 13.5, with points being deducted for overconsumption of salt and sweets, and/or for excess energy intake [33,34]. The PNNS-GS and/or mPNNS-GS scores have been investigated in relation to micro- and macro-nutrient intake [35] and anthropometric changes [34], and have been shown to perform in a similar fashion as the DASH diet score, the Mediterranean diet score, the Dietary Guidelines for Americans Index, and the Diet Quality Index-International, with regard to health outcomes [36,37].

All analyses were conducted with SAS (version 9.4, SAS Institute, Inc., Cary, NC, USA), the tests of statistical significance were two-sided, and the significance level was set at 0.05. 

## 3. Results

### 3.1. Sample Characteristics

**Figure 1:**
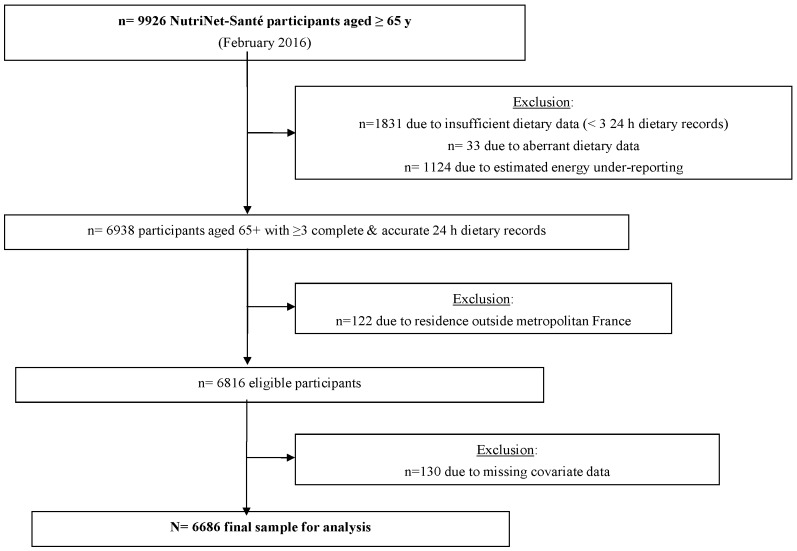
Participant selection flowchart.

By February 2016, there were 9926 individuals aged 65 and older who had enrolled in NutriNet-Santé. From that sample we excluded: 18.4% due to insufficient dietary data (fewer than three 24 h dietary records), 0.3% due to aberrant dietary data, 11.3% due to estimated energy under-reporting, 1.2% due to residence outside metropolitan France, and 1.3% due to missing covariate data. Thus, the final sample available for analysis included *n* = 6686 individuals (Figure 1). Among NutriNet-Santé participants aged 65 and older, those included in the present analysis were somewhat younger, with more years of formal education, less likely to be current smokers, less likely to live alone, and displayed healthier profiles (lower mean BMI, and lower prevalence of hypertension, diabetes, and cardiovascular disease) compared with individuals excluded from the analysis (all *p* < 0.001, data not tabulated). There were no significant differences between included and excluded participants with regard to sex or residential area density. 

The sex-specific sociodemographic characteristics of the sample are presented in Table 2. Almost all (97.1%) participants were retired, 55.3% were women and the mean age was 68.9 (SD = 3.9) years. In total, 9.2% of the participants were aged 75+ years. The mean BMI was 25.1 (SD = 4.1) with 10.5% of the participants having obesity. Half of the sample reported post-secondary education and former smoking, and a third reported prevalent hypertension and residence in a rural or semi-rural area (<20,000 inhabitants). The overall prevalence of diabetes and major cardiovascular disease was low (6.1% and 4.7%, respectively). Based on a mean of 4.8 dietary records per individual, the estimated mean total energy intake in the full sample was 1877.1 (SD = 470.7) kcal/day.

### 3.2. Dietary Patterns in French Elderly

The three DP (Table 1) explained 25% of the total variance in dietary intake in our sample. The first DP had an eigenvalue of 2.18 and explained 10% of the dietary intake variance. It was labeled “healthy” as it featured consumption of fruit, vegetables, nuts, whole grains, fish, vegetable oils, and was low in sugar. The second DP had an eigenvalue of 1.82 and explained an additional 8% of the variance in dietary intake. It was labeled “western” because it was characterized by consumption of red and organ meats, appetizers, cheese, and alcohol. The third DP had an eigenvalue of 1.41 and explained an additional 6% of the variance in intake. It was labeled “traditional” and represented consumption of bread, potatoes, milk, vegetables, butter/margarine, and stock. 

### 3.3. Sociodemographic and Health Status Correlates of the Three DP

Most of the performed tests for interaction by sex (with total energy and protein intake as the respective exposure variables) were statistically significant. Next, given the very low variability with regard to retirement status, prevalent diabetes, and history of major cardiovascular disease (especially among women), these covariates were not retained in the multivariable models. The sex-specific associations of each DP with the remaining sociodemographic and health status correlates are presented in Table 3. The “healthy” DP was positively associated with post-secondary education and living alone (in men and women), and being a former smoker (in women). It was negatively associated with being overweight (in men and women), current smoking (in men), age 75+ years, hypertension, and obesity (in women). Overall, the strongest correlates of the “healthy” DP in terms of absolute value were current smoking in men and obesity in women. The “western” DP was positively associated with BMI (in men) and being a former or current smoker across sex. It was negatively associated with age 75+ years (in women), and living alone (in men and women). The “traditional” DP was positively associated with age and negatively associated with being a former or current smoker, and residing in an urban or semi-urban area, across sex. In addition, it was negatively associated with post-secondary education only in men. For both the “western” and “traditional” DP, the strongest correlate in terms of absolute value in both men and women was current smoking. 

### 3.4. Supplementary Analysis

The results of the supplementary analysis are presented in Supplementary Table S2. Overall, the mean mPNNS-GS a priori score was slightly lower in men than in women (8.3 ± 1.6 versus 8.7 ± 1.6), and the score range in the full sample was from 1.3 to 13.3 points.

## 4. Discussion

In this cross-sectional study we identified three DP and their sociodemographic and health status correlates using a large nationwide sample of elderly individuals recruited from the general French population. The “healthy” DP (marked by consumption of fruit, vegetables, whole grains, fish, nuts, and vegetable oils) exhibited the most sex-specific differences, whereas the “traditional” DP (characterized by consumption of bread, potatoes, milk, vegetables, butter/margarine, and stock) was the most uniform across sex. Interestingly, while rich in plant-based foods, the “healthy” DP was not necessarily low-fat, as it included vegetable oils and nuts. In turn, food from animal sources was featured in both the “traditional” and the “western” DP, the latter also being marked by alcohol consumption. Overall, the DP identified in the present study and their diversity resembled those observed in some prior studies with elderly as well as middle-aged individuals in French and international settings [18,20,21,38].

To the best of our knowledge, this is the only available study providing up-to-date information about DP and their sociodemographic correlates using a nationwide sample of French elderly individuals. A preliminary report based on a small sample of elderly women from a single French region recently identified four DP: (1) fish/seafood/fruit/pulses; (2) moderate consumption of vegetables, cereals/starchy food/dairy; (3) pizza/fast food; and (4) dairy/fruit/meat/cereal/vegetables [22]. Whereas there was overlap between the DP identified in the present study and those in the preliminary report, the latter did not find any significant associations between DP and age, marital status, or educational level [22]. 

Across sex, increasing age was positively associated with adherence to the “traditional” DP, consistent with prior findings among European (including French) elderly [15,32]. For example, prior research within the EPIC-Elderly cohort had revealed that age was positively associated with preference for a sweet- and fat-dominated diet, whereas younger age was associated with preference for a plant-based diet [15]. Also, prior research with French elderly had shown that increasing age was associated with reduced consumption of meat, cereals, fish, vegetables, and pulses [17].

In the present analysis, men with post-secondary education were less likely to adhere to the “traditional” DP while no association was found among women. Likewise, education did not play a role in the “western” DP, yet it was positively correlated with the “healthy” DP across sex, thus partly supporting the main study hypothesis. Previous findings among French elderly have also demonstrated that the proportion of regular consumers of fish, raw and cooked vegetables, and fruit (features of the “healthy” DP) was positively associated with education [17]. Findings among elderly from the EPIC cohort have likewise shown that following a predominantly plant-origin or “prudent” diet was positively associated with education [39] whereas a sweet- and fat-dominated diet exhibited an inverse association [15]. Overall, a higher educational level has been associated with “healthy” DP in international settings [21,40]. 

In addition to advancing age and a low level of education, being widowed or living alone has been highlighted as a major risk factor for nutritional deficiencies [17,30]. Contrary to our expectations, living alone was positively associated with adherence to the “healthy” DP and inversely associated with the “western” DP in both men and women. Because the “healthy” DP included foods that arguably do not require lengthy preparation (raw vegetables, fruit, nuts), it is possible that individuals living alone might have a propensity to consume salads and other easy dishes. We could also speculate that elderly individuals living alone might be more likely to take advantage of meal delivery services compared with their cohabiting counterparts. In fact, literature reviews have documented a number of advantages of home-delivered meal programs, including improved diet quality, reduced food insecurity and nutritional risk, socialization opportunities, improvement in dietary adherence, and overall positive effects of quality of life [41]. Recently published findings among Canadian elderly also revealed that living alone was associated with a “healthy” DP [21].

Next, smoking status emerged as the strongest correlate of the three DP in terms of absolute value. It was inversely associated with the “traditional” DP and the “healthy” DP (in men only), and positively associated with the “western” DP (which features alcohol consumptions) across sex. Prior European studies with the elderly had shown that the highest percentage of non-smokers was found among those adhering to a “healthy” or “prudent” diet [42]. Likewise, in the EPIC-Elderly cohort, adhering to a plant-origin diet was associated with never or past smoking [15], whereas in the EPIC-E3N cohort, current and former smoking were positively associated with a DP characterized by meat and alcohol consumption [18]. Overall, such findings underscore the clustering of health/risk behaviors [43].

In the current obesogenic environment, the role of overweight and obesity in dietary behaviors is of particular interest. The alarmingly high prevalence of obesity and diet-related chronic diseases in industrialized countries is in fact regarded as the result of the dietary transition entailed by the globalization processes [1]. It has been suggested that adhering to a healthy diet, such as the Mediterranean diet, is currently decreasing owing to environmental and lifestyle changes provoked by economic and globalization factors [44]. In the present sample, over a third of women and over half of men were either overweight or obese. Overweight displayed a negative association with the “healthy” DP in both men and women, whereas being obese had a negative association with that DP only among women. In turn, both overweight and obesity displayed a positive association with the “western” DP among men. However, prior research within the EPIC-Elderly cohort had revealed that preference for a plant-origin diet was associated with an increased BMI yet decreased waist-to-hip ratio, whereas preference for a sweet- and fat-dominated diet was associated with decreased BMI [15]. In fact, relative abdominal obesity might be a more informative marker than high BMI among the elderly, especially with regard to morbidity and mortality risk [45]. Finally, we did not observe an association between residential area with either the “healthy” or the “western” DP, which might suggest that the area density might have a lesser impact on the dietary choices of the elderly than on the respective choices among middle-aged adults [32]. 

Because the NutriNet-Santé e-cohort is focused on nutrition, it likely attracted health- and nutrition-conscious volunteers, which is seen as a limitation of the study, bearing on its generalizability. Moreover, a prior study comparing the sociodemographic characteristics of the NutriNet-Santé volunteers with the corresponding national estimates (2009 French Census), revealed convergence regarding the geographical distribution and divergence regarding the sex distribution and educational level, with the cohort including higher proportions of women and relatively well-educated individuals compared with the national figures [46]. Likewise, the proportion of individuals aged 65+ years was over three times smaller in the cohort than in the general population [46]. In turn, despite decreasing sociodemographic disparities, the proportion of elderly Internet users is smaller than the respective proportions of young and middle-aged users. According to French 2014 data, among individuals aged 60–69 years, 76% had Internet access at home (versus 82% in the general population, and 45% among those aged 70+ years) [47]. In addition, 19% of French individuals aged 60–69 years and 56% of those aged 70+ years did not report using the Internet in 2014. Compared with users in those age groups, the non-users were more likely to live alone and to have low/no formal education [47]. Next, a non-negligible proportion of elderly participants (~31%) were excluded from the present analysis due to issues related to data completeness and quality. All of these aspects suggest the potential presence of selection bias and necessitate further caution when extrapolating the findings of the present study. Moreover, the recruitment strategies precluded knowledge of participation and refusal rates. Another limitation was the non-negligible amount of missing data regarding physical activity, hence its omission from the analysis. However, prior research with a representative French sample had revealed that none of five DP was associated with physical activity [32]. Another potential limitation of the DP assessment was related to the fact that participants in NutriNet-Santé had advance knowledge of the assessment days, which might have triggered the potential for reactivity (i.e., reporting intake that is healthier than usual) [48]. Finally, whereas no data on dental status were included in the analysis, authors have suggested that masticatory ability explains only part of the variance in food intake among the elderly [49], and that instead of excluding foods from their menu, individuals might adapt their cooking and food preparation practices in order to overcome chewing problems [50].

Important strengths of the study include the very large sample derived from the general French population via nationwide recruitment, the use of several 24 h dietary records (as opposed to food frequency questionnaires) for the extraction of DP, and the modeling of a large number of sociodemographic correlates. In turn, a recent comparative study found support for the generalizability of the dietary data in NutriNet-Santé, revealing quantitative parallels with dietary intake data provided via dietitian interviews in a representative population sample [51]. Next, we modeled a posteriori factor analysis derived DP, which are population-specific. Whereas the decisions about food group and DP retention are inherently subjective [52], factor analysis permits the differentiation of unique and shared variance and takes into account measurement (random) error [53]. Even though a posteriori DP have the potential to account for the quantity of intake and for the synergy among dietary/nutrient components, and likely reflect overall eating habits [54], they are nonetheless exploratory in nature and do not permit any direct comparisons with DP derived in other French or international studies. Therefore, in a supplementary analysis, we calculated an a priori score (mPNNS-GS) in order to augment understanding of the DP in our sample with respect to adherence to dietary guidelines, and to permit cross-study comparisons. Indeed, in a previous French study (Etude SU.VI.MAX), the following mPNNS-GS cutoffs for low, moderate, and high adherence to dietary recommendations were defined: <5.5 points, 5.5–8.5 points, and >8.5 points [34].

Overall, global-scale monitoring of dietary practices is critical for the development of well-targeted public health efforts aimed at reducing the incidence of noncommunicable diseases including obesity [2]. An examination of diet trends among the elderly over the past three decades revealed an increase in total calories consumed, with bread and desserts becoming dominant calorie sources [55]. The present cross-sectional study provides up-to-date information about the diversity of DP among European elderly, underscoring important sex-specific differences. The “healthy” DP, rich in fruit, vegetables, whole grain products, vegetable oils, nuts, and fish without being low-fat, explained the largest amount of the variance in intake in the sample. Future studies could augment the findings by investigating additional correlates of DP, such as dental status and sensory capacities, and by replicating the models in longitudinal and international contexts. Finally, the present study could help inform future investigations within the ongoing ALIMASSENS Collaborative Project.

## Figures and Tables

**Table 1:** Factor loadings of 22 food and beverage groups onto three dietary patterns (Etude NutriNet-Santé).

	Pattern 1: Healthy	Pattern 2: Western	Pattern 3: Traditional
Food			
Vegetables	**0.48**	**−0.28**	**0.44**
Fresh fruit	**0.47**	−0.02	0.12
Dried fruits, nuts	**0.46**	−0.01	−0.02
Fish	**0.28**	−0.08	−0.05
Whole-grain products	**0.51**	−0.12	−0.13
Vegetable oils	**0.45**	0.06	0.08
Milk-based desserts	**−0.29**	−0.05	0.09
Bread, toast	**−0.27**	**0.37**	**0.51**
Broth, stock	0.07	**−0.33**	**0.61**
Butter, margarine	−0.02	0.13	**0.60**
Potatoes, tubers	0.01	0.08	**0.50**
Sweet products (honey, candy)	0.05	0.22	**0.37**
Appetizers	0.05	**0.39**	−0.08
Cheese	0.16	**0.52**	0.22
Cold cuts	−0.03	**0.48**	0.03
Red meat, organ meats	−0.23	**0.27**	0.16
Pastries, high-fat and high-sugar products	−0.04	**0.29**	0.05
Yogurt	0.12	**−0.38**	−0.06
Legumes/pulses	0.22	−0.04	0.11
Beverages			
Milk	**−0.28**	−0.12	**0.27**
Non-alcoholic non-sweetend beverages	**0.48**	−0.01	−0.00
Alcoholic beverages	−0.04	**0.64**	−0.07

**Table 2:** Baseline characteristics of participants aged ≥65 years (Etude NutriNet-Santé, *n* = 6686) ^a^.

	Men (*n* = 2991)	Women (*n* = 3695)	*p* ^b^
Age, years, mean (SD)	69.2 (4.0)	68.6 (3.8)	<0.0001
65–69 years	1856 (62.1)	2551 (69.0)	
70–74 years	822 (27.5)	842 (22.8)	
≥75 years	313 (10.5)	302 (8.2)	
Body mass index, kg/m^2^, mean (SD)	25.6 (3.5)	24.7 (4.5)	<0.0001
Normal, 18.5–24.9	1423 (47.6)	2247 (60.8)	
Overweight, 25.0–29.9	1285 (43.0)	1026 (27.8)	
Obese, ≥30	283 (9.5)	422 (11.4)	
Educational level			<0.001
Up to high school	1387 (46.4)	2007 (54.3)	
Post-secondary education	1604 (53.6)	1688 (45.7)	
Living alone	370 (12.4)	1337 (36.2)	<0.0001
Retired	2925 (97.8)	3564 (96.5)	0.001
Most recent occupation			<0.0001
Farmer, manual labor, blue-collar	259 (8.7)	1053 (28.5)	
Office staff, self-employed	1887 (63.1)	1228 (33.2)	
Professional/executive staff	845 (28.3)	1414 (38.3)	
Smoking status			<0.0001
Never	848 (28.4)	2055 (55.6)	
Former	1949 (65.2)	1448 (39.2)	
Current	194 (6.5)	192 (5.2)	
Residence in urban area (≥20,000 inhabitants)	1841 (61.6)	2403 (65.0)	0.003
Hypertension ^c^	1254 (42.0)	1119 (30.7)	<0.0001
Diabetes ^d^	273 (9.2)	136 (3.7)	<0.0001
Cardiovascular disease history ^e^	224 (7.5)	92 (2.5)	<0.0001
Total dietary energy, kcal/day, mean (SD) ^f^	2122.6 (473.1)	1678.4 (362.5)	<0.0001

Values refer to number (%) except when noted otherwise. ^a^ Eligibility: residence in metropolitan France and ≥3 complete 24 h dietary records provided during the first two years after enrollment; ^b^ *p*-values obtained from chi-squared tests and Student *t* tests, as appropriate; ^c^ Prevalent hypertension based on self-report and/or report of antihypertensive treatment; ^d^ Prevalent diabetes type 1 or type 2 based on self-report and/or report of anti-diabetic treatment; ^e^ Prior myocardial infarction, stroke, or acute coronary syndrome; ^f^ Calculated from all available 24 h dietary records provided during the first two years after enrollment.

**Table 3:** Sex-specific associations of each dietary pattern with several sociodemographic and health status correlates (Etude NutriNet-Santé, *n* = 6686).

	Dietary Pattern 1				Dietary Pattern 2				Dietary Pattern 3			
	Healthy				Western				Traditional			
	Men		Women		Men		Women		Men		Women	
	beta	*p*	beta	*p*	beta	*p*	beta	*p*	beta	*p*	beta	*p*
Age, years ^a^												
70–74 years	0.05	0.28	−0.03	0.44	−0.06	0.09	−0.05	0.08	0.12	0.003	0.13	<0.0001
75+ years	−0.03	0.64	−0.15	0.004	−0.09	0.12	−0.10	0.03	0.25	<0.0001	0.30	<0.0001
Body mass index, kg/m^2^ ^b^												
Overweight, BMI: 25.0–29.9	−0.19	<0.0001	−0.10	0.003	0.18	<0.0001	0.03	0.32	−0.03	0.46	0.03	0.34
Obesity, BMI ≥ 30	−0.11	0.13	−0.21	<0.0001	0.13	0.03	0.04	0.28	0.03	0.62	0.08	0.06
Post-secondary education	0.16	<0.0001	0.08	0.005	−0.01	0.69	−0.01	0.79	−0.10	0.004	0.00	0.97
Living alone	0.18	0.002	0.11	0.0002	−0.11	0.02	−0.14	<0.0001	−0.08	0.14	−0.01	0.82
Smoking ^c^												
Former smoker	0.03	0.52	0.10	0.0009	0.29	<0.0001	0.15	<0.0001	−0.22	<0.0001	−0.21	<0.0001
Current smoker	−0.36	<0.0001	−0.07	0.30	0.67	<0.0001	0.27	<0.0001	−0.43	<0.0001	−0.27	<0.0001
Hypertension	0.02	0.70	−0.09	0.007	0.01	0.69	−0.00	0.94	−0.05	0.15	0.05	0.07
Urban area (≥20,000 inhabitants)	−0.02	0.58	0.01	0.73	0.02	0.51	0.01	0.62	−0.16	<0.0001	−0.09	0.0009

Results from multivariable linear regression models adjusted for the listed covariates. ^a^ Reference = age 65–69 years; ^b^ Reference = normal weight, BMI: 18.5–24.9 kg/m^2^; ^c^ Reference = never smoker.

## Supplementary Materials

The following are available online at http://www.mdpi.com/2072-6643/8/8/484/s1, Table S1: Intake in grams/day (Etude NutriNet-Santé, *n* = 6686), Table S2: mPNNS-GS score in the full sample and by sex (Etude NutriNet-Santé, *n* = 6686).
