# Supplementary material for: Sex-Specific Sociodemographic Correlates of Dietary Patterns in a Large Sample of French Elderly Individuals
Source: Nutrients. 2016 Aug 8;8(8):484. doi: 10.3390/nu8080484 (PMC4997397; doi:10.3390/nu8080484)
Supplement: Supplementary file 1 [file nutrients-08-00484-s001.docx]

Supplementary Materials: Sex-Specific Sociodemographic Correlates of Dietary Patterns in a Large Sample of French Elderly Individuals

Valentina A. Andreeva, Benjamin Allès, Gilles Feron, Rebeca Gonzalez, Claire Sulmont-Rossé, Pilar Galan, Serge Hercberg and Caroline Méjean

**Table S1.** Intake in grams/day (Etude NutriNet-Santé, *n* = 6686).

| **Food Group** | **Mean** | **SD** |
| --- | --- | --- |
| Red meat, organ meats | 49.36 | 38.69 |
| Cold cuts | 27.38 | 26.29 |
| Poultry | 24.60 | 28.61 |
| Fish | 41.59 | 37.59 |
| Seafood | 9.83 | 19.40 |
| Eggs | 16.03 | 19.84 |
| Milk | 77.42 | 121.41 |
| Cheese | 35.92 | 26.44 |
| Yogurt | 92.61 | 85.62 |
| Milk-based desserts | 25.76 | 42.84 |
| Bread, toast | 85.33 | 71.14 |
| Pasta | 24.78 | 33.31 |
| Rice | 14.23 | 25.50 |
| Other non-breakfast cereals | 10.81 | 21.99 |
| Potatoes, tubers | 56.09 | 49.42 |
| Breakfast cereal | 4.54 | 13.67 |
| Whole-grain foods | 41.71 | 54.01 |
| Legumes | 12.80 | 23.88 |
| Biscuits | 5.89 | 10.97 |
| Cakes, pastries | 27.01 | 35.01 |
| High fat/sugar foods | 12.99 | 19.35 |
| High-sugar foods | 25.50 | 23.43 |
| Appetizers | 2.84 | 7.52 |
| Vegetables | 258.05 | 118.37 |
| Fruit | 249.69 | 152.15 |
| Dried fruits | 3.63 | 10.89 |
| Nuts, oleaginous fruits | 6.52 | 13.58 |
| 100% fruit or vegetable juice | 39.46 | 65.63 |
| Vegetable oils | 9.55 | 8.87 |
| Butter, margarine | 11.70 | 10.04 |
| Other fats | 23.46 | 17.35 |
| Broth, stock | 47.69 | 55.77 |
| Non-alcoholic, unsweetened beverages | 988.27 | 463.84 |
| Non-alcoholic, sweetened beverages | 17.71 | 54.11 |
| Alcoholic drinks | 131.54 | 158.01 |

**Table S2.** mPNNS-GS score in the full sample and by sex (Etude NutriNet-Santé, *n* = 6686).

|  | **Full Sample** | **Men** | **Women** |
| --- | --- | --- | --- |
| Mean | 8.48 | 8.26 | 8.65 |
| SD | 1.62 | 1.60 | 1.62 |
| Min score | 1.29 | 1.29 | 2.83 |
| Median | 8.55 | 8.30 | 8.75 |
| Max score | 13.30 | 12.80 | 13.30 |

mPNNS-GS, modified Programme National Nutrition Santé-Guideline Score.
